# Long non‐coding RNA linc00261 suppresses gastric cancer progression *via* promoting Slug degradation

**DOI:** 10.1111/jcmm.13035

**Published:** 2016-11-23

**Authors:** Yingcong Yu, Linjin Li, Zhiqiang Zheng, Senrui Chen, Ende Chen, Yiren Hu

**Affiliations:** ^1^ Department of GastroenterologyThe Third Clinical College of Wenzhou Medical UniversityWenzhou People's HospitalWenzhouChina; ^2^ Department of Urology SurgeryThe Third Clinical College of Wenzhou Medical UniversityWenzhou People's HospitalWenzhouChina; ^3^ Department of General SurgeryThe Second Affilated Hospital of Wenzhou Medical UniversityWenzhouChina; ^4^ Department of General SurgeryThe Third Clinical College of Wenzhou Medical UniversityWenzhou People's HospitalWenzhouChina

**Keywords:** long non‐coding RNA, gastric cancer, epithelial–mesenchymal transition, Slug, invasion

## Abstract

Gastric cancer (GC) remains a threat to public health with high incidence and mortality worldwide. Increasing evidence demonstrates that long non‐coding RNAs (lncRNAs) play critical regulatory roles in cancer biology, including GC. Previous profiling study showed that lncRNA linc00261 was aberrantly expressed in GC. However, the role of linc00261 in GC progression and the precise molecular mechanism remain unknown. In this study, we report that linc00261 was significantly down‐regulated in GC tissues and the expression level of linc00261 negatively correlated with advanced tumour status and clinical stage as well as poor prognostic outcome. *In vitro* functional assays indicate that ectopic expression of linc00261 suppressed cell invasion by inhibiting the epithelial–mesenchymal transition (EMT). By RNA pull‐down and mass spectrum experiments, we identified Slug as an RNA‐binding protein that binds to linc00261. We confirmed that linc00261 down‐regulated Slug by decreasing the stability of Slug proteins and that the tumour‐suppressive function of linc00261 can be neutralized by Slug. linc00261 may promote the degradation of Slug *via* enhancing the interaction between GSK3β and Slug. Moreover, linc00216 overexpression repressed lung metastasis *in vivo*. Together, our findings suggest that linc00261 acts a tumour suppressor in GC by decreasing the stability of Slug proteins and suppressing EMT. By clarifying the mechanisms underlying GC progression, these findings may facilitate the development of novel therapeutic strategies for GC.

## Introduction

GC represents the fourth most frequent cancer and second leading cause of cancer‐related deaths worldwide, with a high mortality 1. The majority of the patients suffering from GC are diagnosed at advanced stages accompanied with extensive invasion, lymph node and distant metastasis 2, 3. Despite the recent advances the surgical techniques and medical treatment of GC, the prognosis of patients with GC remains relatively poor 3. Recurrence and metastasis are the main causes accounting for the poor prognosis 2, 3. Although remarkable progress has been made in clarifying the molecular mechanism of GC 4, 5, 6, the precise molecular mechanisms of tumourigenesis remain largely unknown. Thus, the identification of new mechanisms associated with GC pathogenesis is vital for the development of effective targeted treatment of GC.

The genome sequencing projects have revealed that the human genome is comprised of less than 2% protein‐coding genes, and more than 90% of the genome is transcribed as non‐coding RNAs (ncRNAs) 7. non‐coding RNAs can be can be broadly categorized into two groups: short non‐coding RNAs and lncRNAs, depending on their length. lncRNAs are defined as a class of non‐coding RNA transcripts longer than 200 nucleotides (nt) with limited protein‐coding potential 7. Accumulating evidence demonstrates that lncRNAs play roles in a variety of biological processes, including chromatin remodelling, cell differentiation and immune responses 8, 9, 10. Unlike the established mechanism of miRNA and siRNA silencing target genes *via* base‐pairing the mRNA complementary sequences, lncRNAs control the local or global gene expression *via* diversified and complicated manners 11, 12, 13, 14. A growing volume of literature has showed that the dysregulated expression of lncRNAs may paly vital roles in the tumorigenesis of a large number of carcinomas 15, 16, 17.

The lncRNA profiling study 18 revealed that lncRNA linc00261, a lncRNA mapped to 20p11.21, was found to be down‐regulated in GC tissues compared to normal tissue samples. However, the role of linc00261 in GC progression remains unknown. Recently, ectopic expression of linc00261 was found to decrease cell migration and invasion, leading to the inhibition of metastasis *in vitro* and *in vivo* 19.

In the current study, we demonstrated that the expression of linc00261 was significantly down‐regulated in GC tissues compared with normal adjacent tissues, and low linc00261 expression level correlated with poor patient prognosis. Furthermore, *in vitro* functional assays indicated that linc00261 suppressed invasion and inhibited the EMT. Using an *in vivo* animal model, we revealed that linc00261 exerts tumour‐suppressive effects by inhibiting tumour metastasis. We also revealed that linc00261 exerts tumour‐suppressive activity, at least in part, through reducing Slug protein abundance. These results deepened our understanding of the mechanism underlying GC progression and enabled the development of new therapeutic strategies for GC.

## Materials and methods

### Pharmacological agents

Cycloheximide and MG132 were purchased from Sigma‐Aldrich, St. Louis, Missouri, USA.

### Patient samples

The GC samples and matched adjacent normal tissues were obtained from patients with GC who underwent surgical resection at Wenzhou People's hospital, the third clinical college of Wenzhou Medical University, from November 2009 to December 2013. This study was approved by the Ethics Committee of Wenzhou People's hospital, and written informed consent was obtained from each patient. The study methodologies conformed to the standards set by the declaration of Helsinki. The diagnosis was made by two individual pathologists based on histopathological evaluation, and the corresponding non‐tumorous tissues were >3 cm away from the edge of tumour without obvious tumour cells existence. No anti‐cancer treatments such as radiotherapy or chemotherapy were given before biopsy collection. Tumour staging as performed according to the tumour–node–metastasis (TNM) grading system. All samples were immediately snap‐frozen in liquid nitrogen after surgical resection and then stored at −80°C prior to RNA isolation and qRT‐PCR analysis. Complete clinicopathological data of the patients were available.

### Cell lines

Three GC cell lines (MGC‐803, SGC‐7901, AGS) and a normal gastric epithelium cell line (GES‐1) were purchased from the Institute of Biochemistry and Cell Biology of the Chinese Academy of Sciences (Shanghai, China). The cell lines were cultured in DMEM or RPMI 1640 (Gibco BRL), supplemented with 10% foetal bovine serum (FBS, HyClone) as well as 100 U/ml penicillin and 100 μg/ml streptomycin (Invitrogen, Carlsbad, CA, USA). Cells were maintained in a humidified incubator at 37°C in the presence of 5% CO_2_. All cell lines have been passaged for fewer than 6 months.

### RNA extraction and Quantitative real‐time PCR analysis

Total RNA from GC tissues and cells was extracted using TRIzol reagent (Invitrogen) according to the manufacturer's protocol. The RNA quantity and quality were determined by Nano Drop ND‐2000 spectrophotometer. RNA was reverse transcribed to cDNA by a Reverse Transcription Kit (Takara, Dalian, China). qRT‐PCR was performed on ABI 7500 system (Applied Biosystems, Foster, CA, USA) with standard SYBR‐Green PCR kit protocol (Takara, Japan) according to the manufacturer's instructions. Glyceraldehyde‐3‐phosphate dehydrogenase (GAPDH) was measured as an internal control. Each sample was run in triplicate, and the gene expression levels were normalized by GAPDH expression. qRT‐PCR results were analysed and expressed relative to CT (threshold cycle) values, and then converted to fold changes. Cycling conditions were 95°C 10 min. for initial denaturation, followed by 95°C 40 cycles 15 sec. at for denaturation, 60°C 30 sec. for combined annealing and 72°C 30 sec. for primer extension. The primer sequences used in this study were as the followings: linc00261, 5′‐ ACATTTGGTAGCCCGTGGAG ‐3′(forward), 5′‐ TCTTCCCCGGAGAACTAGCA‐3′(reverse); Slug, 5′‐TGCGATGCCCAGTCTAGAAA‐3′ (forward), 5′‐AAAAGGCTTCTCCCCCGTGT‐3′(reverse); GAPDH, 5′‐AGAAGGCTGGGGCTCATTTG‐3′(forward), 5′‐AGGGGCCATCCACAGTCTTC‐3′(reverse); β‐actin, 5′‐CTGGGACGACATGGAGAAAA‐3′(forward), 5′‐AAGGAAGGCTGGAAGAGTGC‐3′(reverse); E‐cadherin, 5′‐GCCCCATCAGGCCTCCGTTT‐3′ (forward), 5′‐ACCTTGCCTTCTTTGTCTTTGTTGGA‐3′ (reverse); Vimentin, 5′‐CCTGAACCTGAGGGAAACTAA‐3′ (forward), 5′‐GCAGAAAGGCACTTGAAAGC‐3′ (reverse).

### Establishment of lncRNA or lncRNA‐shRNA stable cells

Expression vectors encoding linc00261 were purchased from Fulen Gen Company (Guangzhou, China). For stable knockdown of linc00261, cells were transfected with lentiviral constructs encoding two different linc00261 shRNAs or non‐related lncRNA (GenePharma Tech, Shanghai, China). The primer sequence used was as follows: shRNA‐1, GAAAGCTGTAGCCATTCAA (position: 761–779 nt); shRNA‐2, GCAATTAATTCAGGACACT (position: 2979–2997 nt). To obtain cell lines stably expressing linc00261, cells were infected with the LV‐linc00261 and LV‐control viruses. For construction of lentiviral vector expressing human linc00261 gene, linc00261 cDNA was chemically synthesized. All transfected cells were selected with puromycin (1 mg/ml) for 2 weeks. The overexpression and knockdown efficiencies were verified with qRT‐PCR.

Transient transfection was conducted with Lipofectamine 2000 reagent (Invitrogen) according to the manufacturer's instructions. When cell densities reached about 70%, 50 nM siRNA oligos were introduced into cells. The siRNA oligos sequences targeting Slug were as follows: Slug siRNA: 5′‐GGGAAAUAAAUGACUGGAUdTdT‐3′; Negative control (NC): 5′‐UUCUCCGAACGUGUCACGUdTdT‐3′. Forty‐eight hours after transfection, cells were harvested for qRT‐PCR or Western blot analyses.

### Western blot analysis

Cells were lysed in RIPA lysis buffer (Cell Signal Technology, Danvers, MA, USA) supplemented with protease inhibitors (Roche, Basel, Switzland) on ice. The lysates were collected and then subjected to ultrasonication and centrifugation at 9391g. for 15 min. The supernatants were collected, and protein content was determined by Bradford assay. Total proteins (30–50 μg) were separated by 10–12% SDS‐PAGE and blotted onto a nitrocellulose membranes (GE Healthcare, Marlborough, Massachusetts). The membrane was then blocked with 5% non‐fat milk in Tris‐buffered saline solution with Tween (TBS‐T) for 1 hr at room temperature and incubated with specific primary antibodies at 4°C overnight. After three 5‐min. washes in TBS‐T, the membranes were incubated with horseradish peroxidase (HRP)‐conjugated goat anti‐rabbit IgG antibody (1:2000; Abcam, Cambridge, UK) for 4 hrs. Then, the protein bands of interest were visualized with the Odyssey system (LI‐COR, Lincoln, Nebraska USA). The specific primary antibodies used were as follows: anti‐Slug (ab129153; Abcam), anti‐N‐Cadherin (#4061; CST, USA), anti‐E‐Cadherin (#3195; CST, USA), anti‐Vimentin (#5741; CST, USA) and anti‐β‐actin (#12620; CST, USA).

### Measurement of Cell proliferation, cell cycle and apoptosis

Cell proliferation was determined with Cell Counting Kit‐8 (CCK‐8). Cell cycle and apoptosis were determined with flow cytometric analysis.

### Immunofluorescence analysis

Cells were seeded and fixed on 12 × 12 mm glass slides. For membrane staining (E‐cadherin), cells were fixed with chilled methanol for 15 min. For intracellular staining (Vimentin), the cells were fixed with 4% paraformaldehyde and permeabilized by incubation with 0.5% Triton X‐100 for 2 min. The cells were incubated with 5% non‐fat milk for 15 min. at room temperature. After washing with PBS 3 × 5 min., the cells were incubated with specific primary antibody at 4°C overnight. Antibody dilutions of 1:500 were used for E‐cadherin (CST) and Vimentin (CST). The cells were then washed and incubated with Alexa Fluor 633‐conjugated goat anti‐rabbit IgG for 1 hr. The nuclei were then stained with 4,6‐diamidino‐2‐phenylindole (DAPI). Sections were visualized by a LEICA DMI 3000B fluorescence microscope with an original magnification ×200.

### *In vivo* lung colonization assay

All animal experiments were performed in animal laboratory centre of Wenzhou People's Hospital and in accordance with the Guide for the Care and Use of Laboratory Animals published by the US National Institutes of Health (NIH publication no. 85‐23, revised 1996). The study protocol was approved by the Animal Care and Use committee of Wenzhou People's Hospital (approval ID: 2014012). Female or Male nude mice (5‐week‐old) were purchased from the Animal Center of the Chinese Academy of Science (Shanghai, China) and housed in specific pathogen‐free rodent facilities.

SGC7901 cells linc00261 stable overexpression were harvested, washed with PBS and suspended at a density of 1 × 10^7^ cells/ml. A volume of 0.1 ml suspended cells were injected into the tail veins of four mice. Non‐invasive bioluminescence imaging was performed every 2 weeks after injection to quantify the metastasis burden performed with an IVIS 200 Imaging System (Caliper Life Sciences, Hopkinton, Massachusetts). The mice were killed 8 weeks after injection, and the lungs were collected, photographed and visible tumours on the lung surface were counted. The lung tissues were then fixed in 10% formalin and were stained with haematoxylin and eosin. Four mice were used each group.

### Statistical analysis

The statistical analysis was performed with SPSS, Chicago, IL 17.0 software. The data are presented as the means ± standard deviation (S.D.). Continuous variables were compared by Student's *t‐*test or the anova test. If the test result of the variance homogeneity between the groups was significant, the Mann–Whitney *U*‐test was appropriately adopted. In samples with small size (*n* < 30) and with non‐normal distribution and/or elevated dispersion, we also used nonparametric statistics. Two‐tailed *P*‐values less than 0.05 were considered statistically significant.

For a description of other materials and methods used in this study, see the Appendix S1.

## Results

### Linc00261 is a bona fide non‐coding RNA in GC

We found that linc00261 was included in NONCODE2016 [NONCODE: NONHSAT078992], and information from Ensembl shows that linc00261 [Genebank: NR_001558; Ensembl: ENSG00000259974] shows that it localized in Human chromosome 20; *22541191‐22559280*, which has a transcript of about 4912 nt and consists of four exons. We used 5′ and 3′ RACE to map the exact sequence of linc00261 in the cell lines used in this study (Table S3). Northern blot analysis of linc00261 confirmed the expected size (Fig. S1A). To confirm that linc00261 was indeed a non‐coding RNA, we calculated the codon substitution frequency scores (CSFs) for linc00261 using PhyloCSF. The CSF of linc00261 is −67.6715, indicating that it is a non‐coding RNA. We verified that linc00261 was indeed a non‐coding RNA with online protein‐coding potential assessment softwares (Fig. S1B and C) and an *in vitro* translation assay (Fig. S1D). Subcellular fractionation analysis revealed that linc00261 is mainly located in the cytoplasm of GC cells (Fig. S1E). The RNAfold image is presented in Figure S1F.

### Linc00261 was down‐regulated in GC cell lines and GC tissues

**Figure 1:**
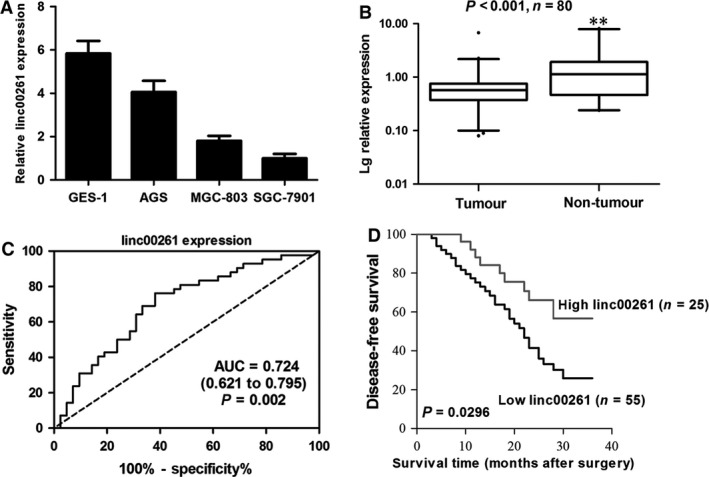
linc00261 expression in GC cell lines, cancer tissues and its clinical significance. (**A**) qRT‐PCR analysis of linc00261 expression levels in gastric cancer cell lines (BGC‐823, MGC‐803 and SGC‐7901) compared with the normal gastric epithelium cell line (GES‐1). Data represent the mean ± S.D. from three independent experiments. (**B**) linc00261 expression was analysed by qRT‐PCR in GC samples and adjacent non‐tumour gastric tissues (*n* = 80). linc00261 expression level was normalized to that of GAPDH. Horizontal lines in the box plots represent the medians, the boxes represent the interquartile range, and the whiskers represent the 2.5th and 97.5th percentiles. The significant differences between samples were analysed using the Wilcoxon signed‐rank test. **P < 0.01. (**C**) ROC curve for prediction of gastric cancer using RT‐qPCR‐based lncRNA‐LEGBC expression level. The AUC was 0.724, with 95% CI and *P* value indicated. (**D**) Kaplan–Meier analysis of disease‐free survival based on linc00261 expression in all the 80 patients.

To investigate the expression pattern of linc00261 in GC, we first determined the expression of linc00261 in diverse GC cell lines performed with qRT‐PCR. Significantly lower linc00261 expression was found in SGC7901, MGC803 and AGS compared with that in GES1 (Fig. 1A). The expression level of linc00261 in GC cell lines was consistent with results from Northern blot analysis. linc00261 expression was also significantly lower in tumour tissues than in adjacent normal tissues (*n* = 80, *P* < 0.01, Fig. 1B), as determined with qRT‐PCR and normalized to GAPDH. Northern blot analysis confirmed that linc00261 was down‐regulated in GC tissues compared to that in adjacent normal tissues (Fig. S2).

According to whether linc00261 expression was up‐ or down‐regulated compared with the corresponding adjacent non‐cancerous tissue samples, the 80 patients with GC were classified into two groups: high‐linc00261 group (*n* = 25) group and low‐linc00261 group (*n* = 55). Lower linc00261 expression levels in GC were significantly correlated with invasion depth (*P* = 0.006) and advanced clinical stage (*P* = 0.003). However, linc00261 expression was not associated with other parameters such as gender (*P* = 0.755) and age (*P* = 0.544; Table S1).

### linc00261 is a diagnostic marker and low linc00261 expression is associated with poor prognosis in patients with GC

We plotted a receiver operating characteristic (ROC) curve with the non‐tumorous tissues adjacent to the tumour tissues as a control. The cut‐off value for predicting GC tissues from normal tissues was 16.57 (Δ Ct value). The area under the ROC curve was 0.724 (95% CI (confidence interval) = 0.621–0.795, *P* = 0.002; Fig. 1C). The sensitivity and specificity were 0.67 and 0.73. Kaplan–Meier survival analysis and log‐rank tests performed with patient postoperative survival were conducted to further evaluate the effects of linc00261 expression and clinicopathological characteristics on the disease‐free survival (DFS) of patients with GC. The results demonstrated that low linc00261 patients had higher recurrence rates (median DFS: 20.8 months) than the high‐linc00261 patients (median DFS: 31.2 months, *P* = 0.0296; Fig. 1D). Univariate analysis identified three prognostic factors: TNM stage; lymph node metastasis; and linc00261 expression level (Table S2). Further analysis in a multivariate Cox proportional hazards model confirmed that TNM stage and linc00261 expression level were significantly associated with DFS (Table S2).

### Effects of linc00261 on GC cells proliferation and cell cycle progression

**Figure 2:**
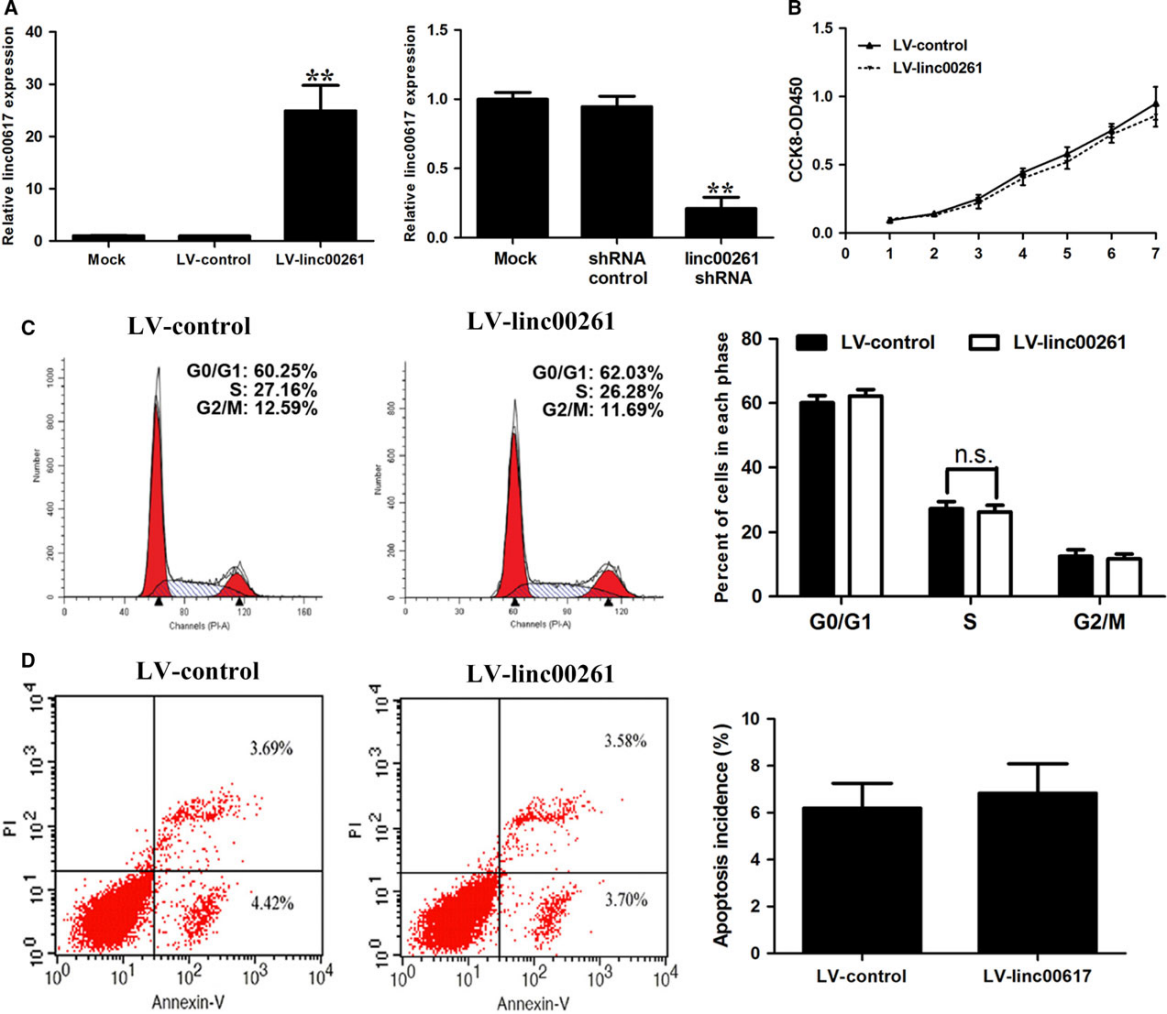
Effect of linc00261 overexpression on gastric cancer cell proliferation, cell cycle and apoptosis. (**A**) The expression of linc00261 (mean ± S.D.) in stable SGC7901 cell clones infected with lentiviruses encoding linc00261. linc00261 expression (mean ± S.D.) in AGS cells that were stably transfected with shRNA against linc00261. (**B**) CCK8 assays were performed to determine the proliferation of SGC7901 cells. (**C**) Cell cycle analysis determined the relative cell numbers in each cell cycle phase after propidium iodide staining of linc00261‐up‐regulated SGC7901 cells. Numbers inside bars represent percentages of cells in each phase. (**D**) Annexin V/PI staining and flow cytometry analysis assessing apoptosis in SGC7901 cells after LV‐linc00261 transfection. Data represent the mean ± S.D. from three independent experiments. N.S., not significant. *P < 0.05; **P < 0.01.

To examine the effect of linc00261 overexpression and knockdown, we performed CCK‐8 assays. As SGC7901 cells harboured the lowest expression level of linc00261 and AGS cells the highest, we selected SGC7901 cells for linc00261 overexpression and AGS cells for linc00261 knockdown. The efficiency of overexpression and down‐regulation was verified by qRT‐PCR (Fig. 2A). CCK‐8 assays revealed that cell growth was not affected in SGC7901 cells with linc00261 overexpression compared with cells transfected with non‐related lncRNA vector (Fig. 2B). We further examined cell cycle progression performed with flow cytometric analysis. The results revealed that linc00261 overexpression had no significant effects on the cell cycle progression (Fig. 2C) and apoptosis (Fig. 2D) of GC cells.

### linc00261 inhibited invasion of GC cells

**Figure 3:**
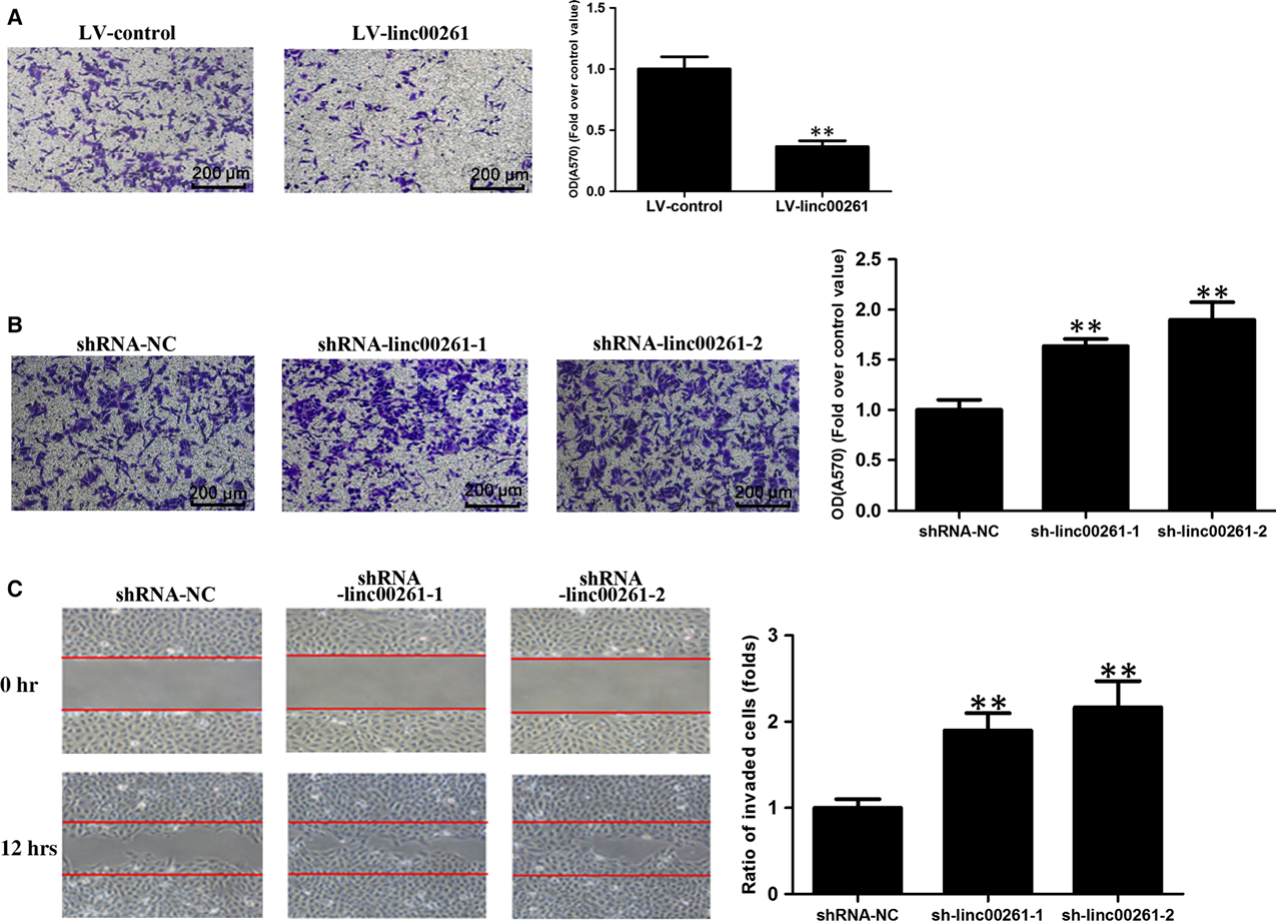
Effect of linc00261 on the invasion of GC cells. (**A**) SGC7901 cells were transfected with LV‐control or LV‐linc00261. Matrigel invasion assay was performed to investigate the invasive ability of SGC7901 cells. Histological analysis of OD (570 nm) absorbance of crystal violet‐stained cells in Matrigel invasion assay. (**B**) AGS cells were transfected with shRNA control or shRNA linc00261. Matrigel invasion assay was performed to investigate the invasive ability of AGS cells. Histological analysis of OD (570 nm) absorbance of crystal violet‐stained cells in Matrigel invasion assay. Data represent the mean ± S.D. from three independent experiments. (**C**) Representative images and quantification of cell motility changes by linc00261 knockdown.*P < 0.05; **P < 0.01.

We also investigated the effects of linc00261 on the invasive ability of GC cells. linc00261‐overexpressing SGC7901 cells showed a significantly lower invasive potential than controls (Fig. 3A). In the meantime, linc00261 knockdown enhanced the invasive ability and motility of AGS cells (Fig. 3B and C).

The EMT is a well‐coordinated process that takes place during embryonic development and a characteristic feature in tumorigenesis 20. During this process, the epithelial phenotype cells lose the expression of E‐cadherin and other components of cell‐to‐cell junctions and adopt a mesenchymal phenotype 21. The EMT process has been demonstrated to be associated with cancer invasion, metastasis and therapeutic resistance 22. We then determined the effect of linc00261 on the EMT process of GC cells.

**Figure 4:**
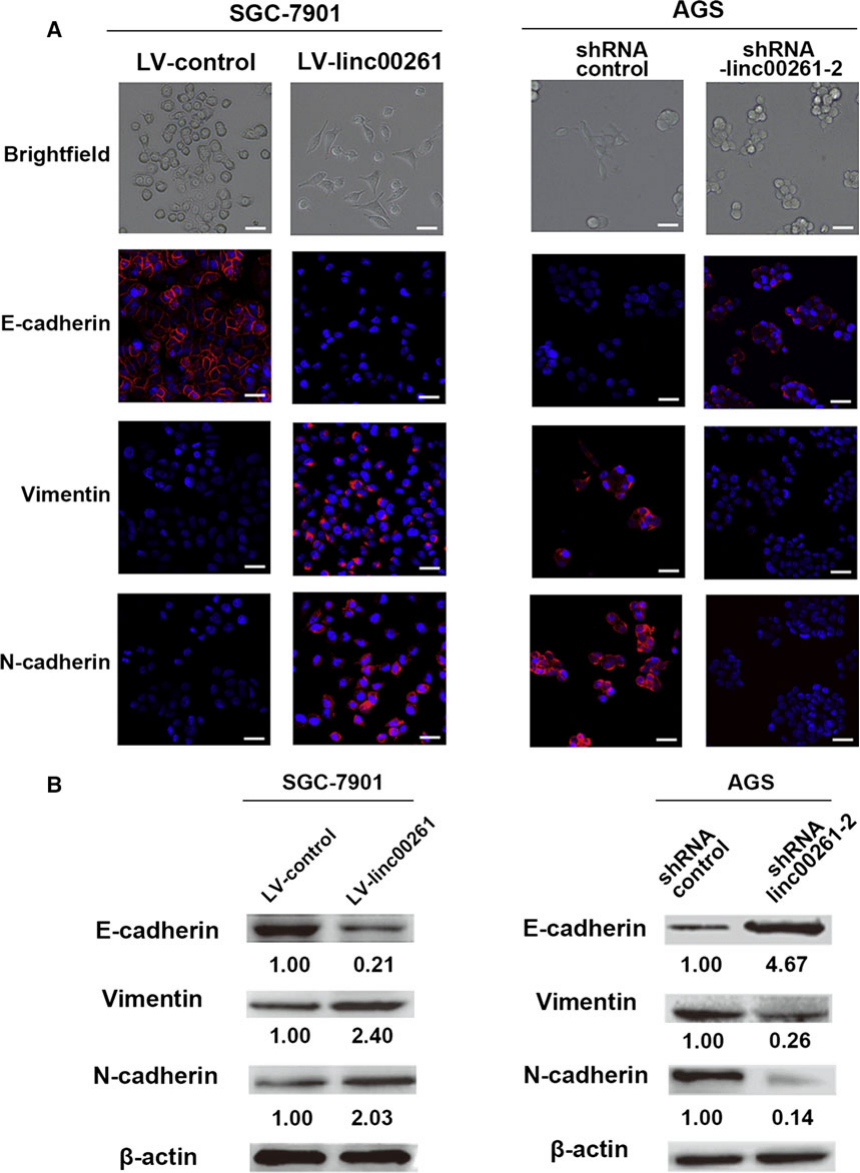
linc00261 suppresses EMT in GC cells. (**A**) Immunofluorescence images for EMT markers of linc00261‐overexpressing or control SGC7901 cells. Scale bar, 20 μm. Western blot analysis of phenotypic markers after linc00261 overexpression (SGC7901). (**B**) Immunofluorescence images for EMT markers of linc00261‐knockdown or control AGS cells. Scale bar, 20 μm. Western blot analysis of phenotypic markers after linc00261 silencing (AGS). Relative protein expression was identified (*n* = 3) and normalized to β‐actin. Data represent the mean ± S.D.

The immunofluorescence analysis demonstrated that ectopic expression of linc00261 in SGC7901 cells leads to a significant up‐regulation of membranous epithelial marker E‐cadherin and a great reduction in the expression of cytoplasmic mesenchymal markers Vimentin (Fig. 4A), while knockdown of linc00261 induced the opposite effects (Fig. 4B). The Western blot analysis confirmed the results from the immunofluorescence analysis (Fig. 4A and B).

### linc00261 suppresses tumour metastasis *in vivo*

**Figure 5:**
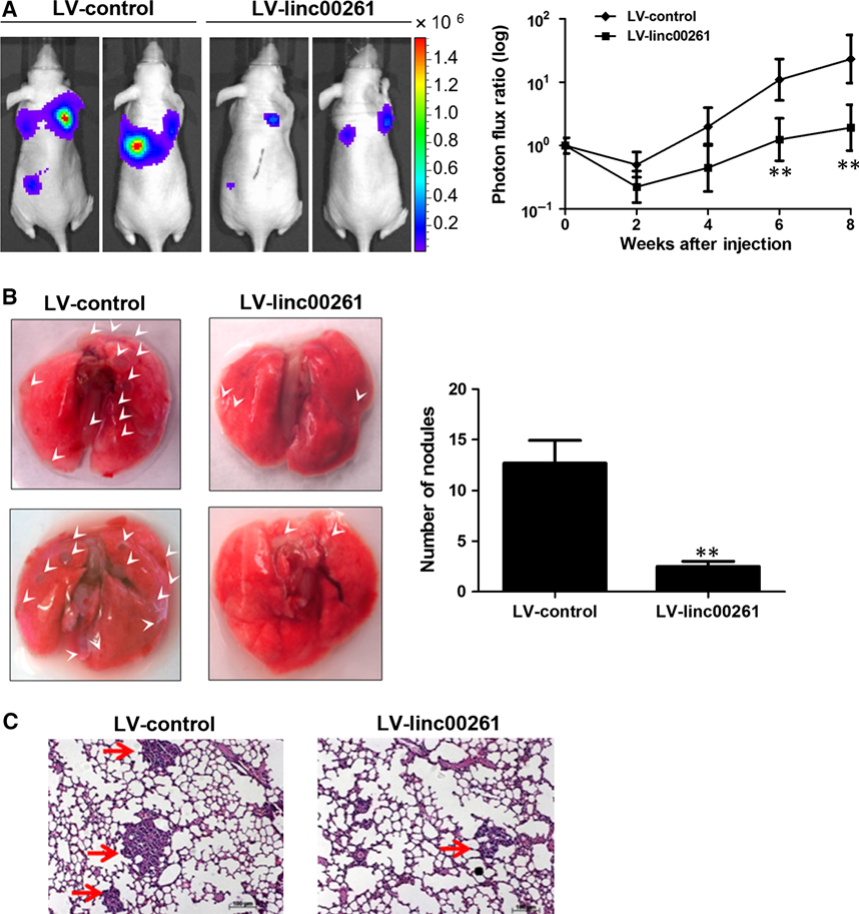
linc00261 suppresses lung metastasis *in vivo*. (**A**) Representative BLI plots of lung metastases in mice after injecting SGC7901 cells stably expressing linc00261 or a control vector; four mice were used each group. (**B**) Lungs were extracted at 8 weeks after tail vein injection, and the number of nodules was counted. Arrow indicated tumours on the surface of the lungs. (**C**) Haematoxylin and eosin‐stained images of lung tissue isolated from nude mice that received intravenous tail injections of SGC7901 cells stably expressing linc00261 or a control vector. Arrows indicate the metastasis nodules (original magnification ×100). Scale bars, 50 μM. Data represent the mean ± S.D. from three independent experiments. *P < 0.05; **P < 0.01. Arrows indicate the metastasis nodules.

To further confirm the tumour‐suppressive function of linc00261, we performed an *in vivo* tail vein metastasis assay using nude mice. We found that linc00261‐overexpressing SGC7901 cells exhibited reduced metastases in the lung, implying that linc00261 may be negatively affecting the extravasation of GC cells (Fig. 5A). Continued BLI monitoring revealed a further reduction in metastatic outgrowth in the lungs of animals injected with linc00261‐overexpressing cells (Fig. 5A). Furthermore, histological analysis confirmed the decrease in the number of metastatic lesions produced by the SGC7901 linc00261‐overexpressing cells compared with control cells (Fig. 5B and C).

### linc00261 binds to Slug and enhances Slug degradation

**Figure 6:**
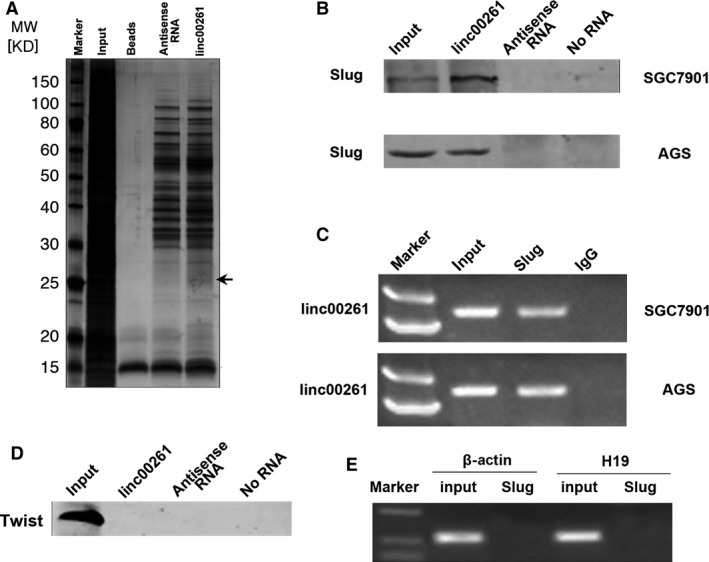
linc00261 binds to Slug protein. (**A**) Representative SDS‐PAGE gel after Coomassie blue staining shows a prominent band at about 25 kD, representing mainly Slug proteins (see also supplemental Table 4). (**B**) RNA pull‐down assay performed in SGC7901 and AGS cells. Biotinylated linc00261 or antisense RNA was incubated with cell extracts, targeted with streptavidin beads and washed, and the associated proteins were resolved on a gel. Western blot analysis detected the specific association of Slug and linc00261 (*n* = 3). (**C**) RIP experiments were performed using the Slug antibody for immunoprecipitation (IP) and a primer to detect linc00261. RIP enrichment was determined relative to the input controls. (**D**) No interaction between linc00261 and Twist could be detected. RNA pull‐down assay performed in SGC7901 cells. Biotinylated linc00261 or antisense RNA was incubated with cell extracts, targeted with streptavidin beads and washed, and the associated proteins were resolved on a gel. Western blot analysis detected the specific association of Twist and linc00261. (**E**) RIP experiments were performed using Slug to immunoprecipitate RNA and a primer to detect H19 and β‐actin.

Recent studies have suggested that lncRNAs participate in molecular regulation pathways through interacting with proteins 8. To further investigate the underlying mechanism by which linc00261 inhibited EMT, we performed an RNA pull‐down assay followed by LC‐MS analysis to identify linc00261‐associated proteins (Fig. 6A). The distinct band specific to linc00261 was excised and subjected to mass spectrometry (Table S4). Slug was detected by Western blotting from three independent RNA pull‐down assays in cell extracts from SGC7901 and AGS cells (Fig. 6B). To further confirm the specific binding between linc00261 and Slug, an RNA immunoprecipitation (RIP) assay was performed using an antibody against Slug. Consistent with RNA pull‐down results, a significant higher enrichment of linc00261 was observed using the anti‐Slug antibody when compared with a nonspecific IgG control (Fig. 6C). Moreover, RNA pull‐down results showed that linc00261 could not specifically bind to Twist (Fig. 6D). No significant enrichments of β‐Actin or H19 with Slug were observed (Fig. 6E).

**Figure 7:**
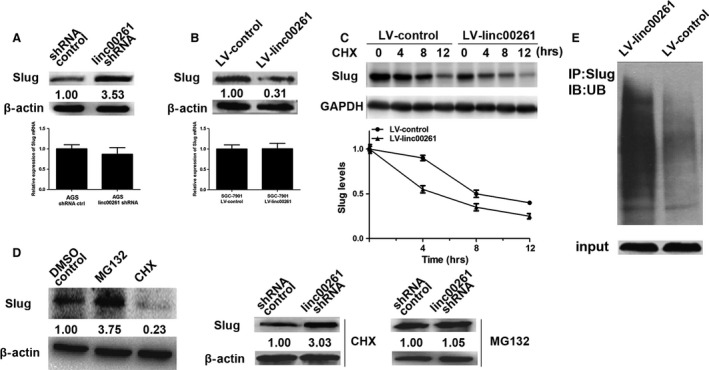
linc00261 decreases the protein level of Slug by inhibiting its stability. (**A**) The protein levels of Slug were detected in linc00261‐knockdown AGS cells by Western blot analysis. The mRNA levels of Slug were detected in linc00261‐knockdown AGS cells by qRT‐PCR (*n* = 3). (**B**) The protein levels of Slug were detected in linc00261‐up‐regulated SGC7901 cells. The mRNA levels of Slug were detected in linc00261‐overexpressing SGC‐7901 cells by qRT‐PCR (*n* = 3). (**C**) linc00261 overexpression enhanced Slug protein degradation. SGC‐7901 cells were transfected with Luc or linc00261. After treating the cells with cycloheximide (CHX, 0.5 μg/μl) for an indicated time, the expression of endogenous Slug protein was analysed by WB. The band intensity of Slug for each time‐point was quantified by ImageJ and plotted. Experiments were repeated for three times, and a representative experiment is presented. Error bars represent S.D. Every experimental group was compared with the control Lucsi group. (**D**) The comparison and quantification of Slug proteins in AGS cells with and without the protein synthesis inhibitor cycloheximide (CHX, 0.5 μg/μl) or the proteasome inhibitor MG‐132 (5 μM) for 24 hrs. linc00261 stable knockdown AGS cells and control cells were incubated with MG132 (5 μM) or CHX (0.5 μg/μl) for 24 hrs. The levels of Slug proteins were detected by Western blot analysis. (**E**) Stable linc00261 overexpressing SGC7901 cells and control cells were treated with MG132 (10 mM) for 6 hrs. Cell lysates were immunoprecipitated with a Slug‐specific antibody, followed by Western blotting with an antibody to ubiquitin. The bottom panel depicts the input of the cell lysates.

Next, we sought to determine the functional relevance of the association between linc00261 and Slug. We detected a significant up‐regulation of the Slug protein upon linc00261 knockdown in AGS cells. However, we did not observe an increase in Slug mRNA levels. (Fig. 7A) We also found a down‐regulation of the Slug protein in linc00261‐overexpressing SGC7901 cells and no significant decrease in Slug mRNA levels. (Fig. 7B) To further investigate whether linc00261 promoted Slug protein degradation, we overexpressed linc00261 in SGC‐7901 cells and measured the Slug protein half‐lives with the cycloheximide chase assay. As anticipated, when linc00261 was overexpressed, the Slug protein half‐life was decreased from 8 to 4.6 hrs. To determine whether linc00261 reduced Slug protein stability, we treated linc00261‐down‐regulated AGS and control cells with the protein synthesis inhibitor cycloheximide (CHX) or the proteasome inhibitor MG‐132. The DMSO was used as the negative control agent. As shown in Figure 7D, CHX decreased the expression of Slug proteins and MG‐132 up‐regulated the expression of Slug proteins. Then, we treated linc00261‐down‐regulated AGS cells with CHX or MG‐132. We found that only MG‐132 abolished the up‐regulation of Slug protein levels in linc00261‐down‐regulated AGS cells. We further examined whether linc00261 affects Slug protein stability by performing an ubiquitination assay and found that the Slug ubiquitination level was significantly higher in SGC7901 cells that overexpressed linc00261 relative to control cells (Fig. 7E). These findings indicate that the ubiquitin–proteasome pathway might be required for linc00261‐mediated reduction in Slug protein.

**Figure 8:**
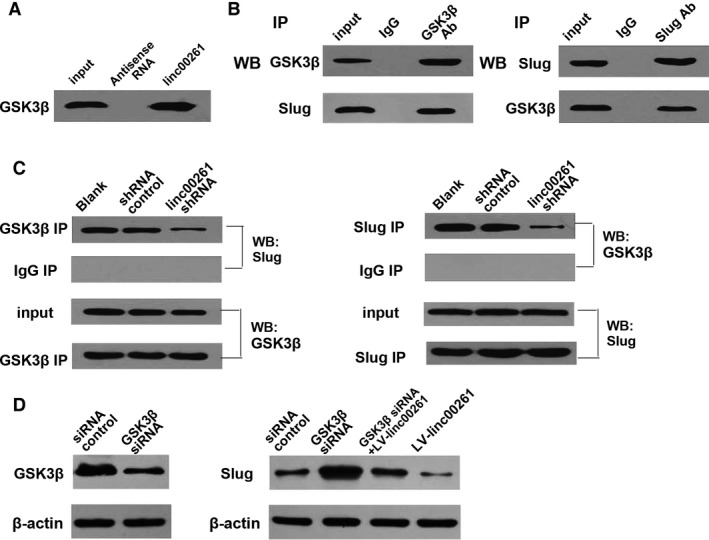
linc00261 promote the degradation of Slug *via* enhancing the interaction between GSK3β and Slug. (**A**) RNA pull‐down assay performed in AGS cells. Biotinylated linc00261 or antisense RNA was incubated with cell extracts, targeted with streptavidin beads and washed, and the associated proteins were resolved on a gel. Western blot analysis detected the specific association of GSK3β and linc00261 (*n* = 3). (**B**) Co‐immunoprecipitation detected the interaction of GSK3β and Slug in AGS cells. The 20% of input (cell lysate) and GSK3β or Slug immunoprecipitates were separated by SDS‐PAGE. The specific immunoprecipitation of GSK3β and Slug was confirmed by Western blot analysis (*n* = 3). (**C**) Immunoprecipitation assay was performed to detect the interaction between GSK3β and Slug after transfection of linc00261 shRNA in AGS cells. (**D**) Western blot analysis confirmed that GSK3β‐specific siRNA down‐regulated the protein level of GSK3β. Knockdown of GSK3β abolished the down‐regulation of protein level of Slug induced by linc00261 overexpression.

Previous studies suggest that Slug phosphorylated by GSK3β undergoes ubiquitination and proteosomal degradation in breast cancer 23 and non‐small cell lung cancer 24. Thus, we have been suggested that linc00261 promote the degradation of Slug *via* enhancing the interaction between GSK3β and Slug. Western blot analysis demonstrated that linc00261 bound specifically to GSK3β (Fig. 8A). We observed that GSK3β interacted with Slug in GC cells (Fig. 8B). Furthermore, knockdown of linc00261 abolished the interaction between GSK3β and Slug (Fig. 8C). Thus, linc00261 is vital for GSK3β and Slug complex interaction. GSK3β‐specific siRNAs were transduced into cells, and the knockdown of GSK3β attenuated the decrease in Slug protein levels induced by linc00261 overexpression (Fig. 8D).

### linc00261 inhibits EMT by suppressing Slug protein

**Figure 9:**
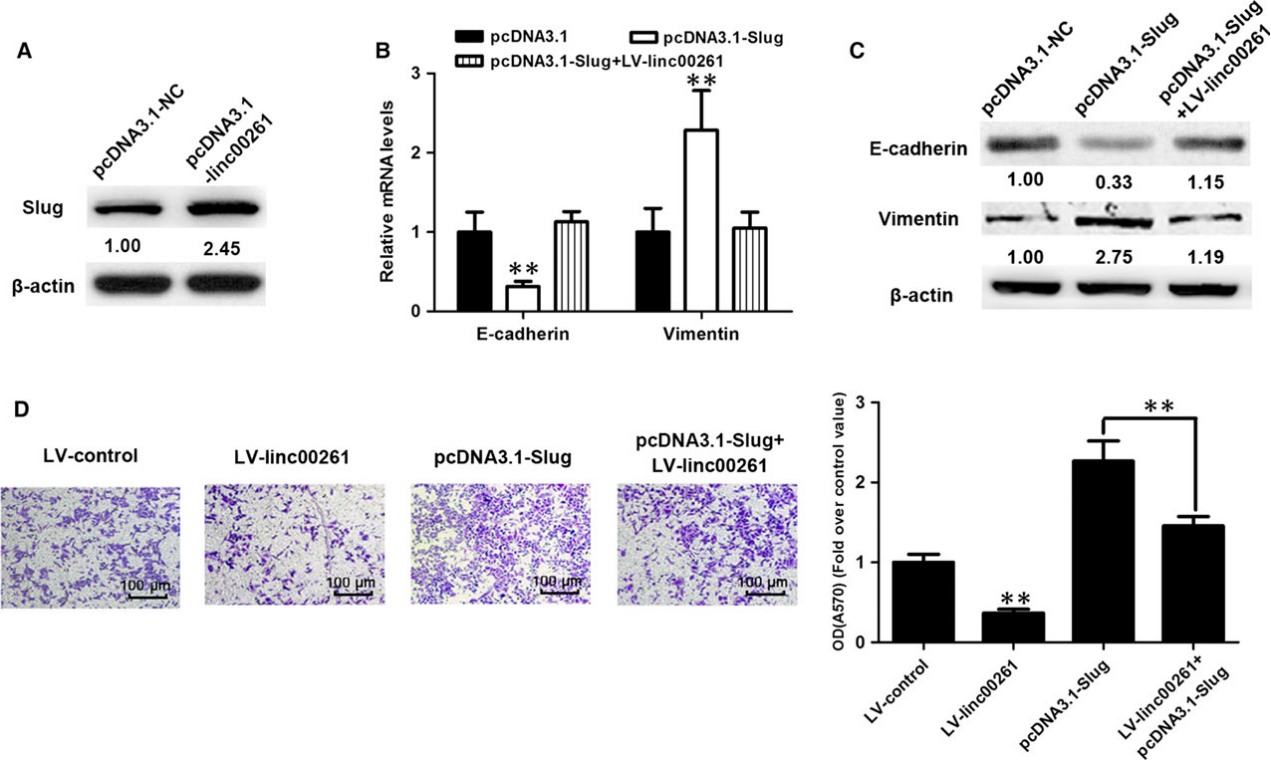
Slug plays a critical role in linc00261‐mediated EMT. (**A**) Western blot analysis was performed to confirm the successful up‐regulation of Slug protein levels. qRT‐PCR analysis (**B**) and Western blots (**C**) of EMT‐specific marker E‐cadherin and Vimentin from SGC‐7901 cells receiving the indicated vectors (**D**) linc00261 overexpression suppressed the invasion of SGC7901 cells and Slug‐overexpression attenuated the linc00261 overexpression‐induced suppression of SGC‐7901 cell invasiveness. Histological analysis of OD (570 nm) absorbance of crystal violet‐stained cells in transwell assay. Data represent the mean ± S.D. from three independent experiments.*P < 0.05. **P < 0.01.

The EMT process is mediated by a set of transcription factors including the zinc‐finger proteins Snai1 and Slug (also known as Snai2), the zinc‐finger/homeodomain proteins ZEB1 and the bHLH factors Twist 22. As a key mediator of EMT, Slug has been demonstrated to be up‐regulated in GC and has been associated with aggressive phenotype of GC 25, 26, 27. Western blot analysis was performed to confirm the successful down‐regulation of Slug protein levels mediated by small interference RNA against Slug (Fig. 9A). As illustrated above, linc00261 suppressed EMT process in GC cells. The expression changes in EMT markers were blunted with Slug knockdown in AGS cells both in the transcript and protein levels (Fig. 9B and C). Functionally, the increase in the invasive potential induced by linc00261 suppression could be attenuated after transfection of Slug siRNA (Fig. 9D).

## Discussion

GC is one of the most common cancers and represents the second leading cause of cancer‐related deaths worldwide 1. Although clinical symptoms are not commonly observed during the early stages of GC development, in most cases, the detection of symptoms during the advanced stage leads to a poor prognosis at the time of diagnosis 2. Despite the recent advances in the management of GC, the prognosis of patients with GC remains relatively poor 3. Recurrence and metastasis are the main causes of the poor prognosis 2, 3. Thus, the exploration of new diagnostic and therapeutic molecular targets for GC is particularly crucial. Recent studies have suggested that many lncRNAs play important roles in GC progression 28, 29, 30. However, the majority of lncRNAs identified in GC have not been investigated extensively.

At present, tumour grade, tumour size and clinical tumour stage are considered to be important predictors of tumour recurrence 31. However, current clinical predictions based on clinicopathological characteristics remain unsatisfactory and molecularly based tumour staging is essential for individualized diagnosis and therapy. The molecular classification of GC has identified a number of protein‐coding genes as valuable biomarkers and prognostic indicators 32, 33, 34. However, a poor overlap exists between these biomarkers of GC. Thus, it might be a better resolution to establish more accurate prognostic gene signatures using a combination of different types of transcripts 35. A growing volume of literature has demonstrated that ncRNAs, predominantly miRNAs, could serve as potential biomarkers of GBC 36, 37. Given the fact that lncRNAs are more abundantly expressed in mammalian cells, it is plausible to speculate that lncRNAs, once regarded as ‘transcriptional noise’, may be potential prognostic indicators in GC.

Previous profiling study identified that linc00261 was down‐regulated in GC tissues compared to normal tissue samples 18. In this study, we confirmed that linc00261 was expressed at a lower level in GC tissues than in adjacent normal tissues. What's more, the expression of linc00261 can discriminate GC from normal tissues. It suggests that linc00261 may play a role in the development of GC. We demonstrated that decreased linc00261 expression levels in GC were significantly correlated with invasion depth, advanced clinical stage and poor prognosis. We also investigated effect of linc00261 on cell proliferation, cell cycle progression and invasion of GC cells by gain‐of‐function and loss‐of‐function experiments. Furthermore, both *in vitro* functional assays and an *in vivo* animal model demonstrated that linc00261 exerts tumour‐suppressive activity by reducing cancer cell invasion ability at least in part *via* suppressing the EMT process.

As a key mediator of EMT, Slug has been demonstrated to be up‐regulated in GC and been associated with aggressive phenotype of GC. The up‐regulated expression of Slug has been shown to be associated with decreased expression of epithelial marker (E‐cadherin) and increased expression of mesenchymal marker (Vimentin) 25, 26, 27. We found that linc00261 binds to Slug protein and promotes the degradation of Slug. Previous studies suggest that GSK3β mediated the ubiquitination and proteosomal degradation of Slug 23, 24. Furthermore, we found that linc00261 promote the degradation of Slug *via* enhancing the interaction between GSK3β and Slug as knockdown of GSK3β attenuated the decrease in Slug protein levels induced by linc00261 overexpression. Furthermore, we found that the EMT with linc00261 knockdown can be blocked by transfection of Slug siRNA, suggesting that linc00261 exhibits tumour‐suppressive activity in GC *via* promoting Slug degradation and inhibiting epithelial–mesenchymal transition.

In conclusion, our study identifies linc00216 as a novel tumour suppressor in GC. The linc00216 was shown to suppress GC development and was possibly a potential therapeutic target for the highly aggressive and malignant GCs in clinical practice.

## Conflict of interest

The authors confirm that there are no conflict of interests.

## Supporting information


**Appendix S1** Materials and methods.
**Table S1** Correlation between linc00261 expression and clinicopathological characteristics of gastric cancer.
**Table S2** Univariate and multivariate analyses of DFS in 80 GC patients by Cox regression analysis.
**Table S3** Linc00261 cDNA sequence.
**Table S4** Mass spectrometry analysis of the proteins pulled down by linc00261.
**Figure S1** (A) Northern blot analysis of linc00261 in GC cells. (B,C) linc00261 is a long non‐coding RNA. Lack of open reading frame in the 4924‐bp linc00261 sense sequence cloned from RACE, as verified by ORF Finder (B) and CPC (C). (D)In the translation assay, a 5598‐bp genomic region containing linc00261 was cloned into a pcDNA vector and expressed using the TnT Quick Coupled Transcription/Translation System (Promega). The absence of a specific band indicated that linc00261 is a transcript with no protein‐coding capacity. Luciferase *in vitro* translation served as positive control. (E) linc00261 was mainly located in the cytoplasm as shown by qRT‐PCR. (F) Prediction of linc00261 structure based on minimum free energy (MFE) and partition function. Color scale indicates the confidence for the prediction for each base with shades of red indicating strong confidence. (http://rna.tbi.univie.ac.at/).
**Figure S2** Expression of linc00261 was determined by northern blotting in 5 normal tissues and 5 paired GC tissues.
