# Supplementary material for: Long non‐coding RNA linc00261 suppresses gastric cancer progression via promoting Slug degradation
Source: J Cell Mol Med. 2016 Nov 23;21(5):955–67. doi: 10.1111/jcmm.13035 (PMC5387161; doi:10.1111/jcmm.13035)
Supplement: Supplementary file 1 — Appendix S1 Materials and methods. Table S1 Correlation between linc00261 expression and clinicopathological characteristics of gastric cancer. Table S2 Univariate and multivariate analyses of DFS in 80 GC patients by Cox regression analysis. Table S3 Linc00261 cDNA sequence. Table S4 Mass spectrometry analysis of the proteins pulled down by linc00261. Figure S1 (A) Northern blot analysis of linc00261 in GC cells. (B,C) linc00261 is a long non‐coding RNA. Lack of open reading frame in the 4924‐bp linc00261 sense sequence cloned from RACE, as verified by ORF Finder (B) and CPC (C). (D)In the translation assay, a 5598‐bp genomic region containing linc00261 was cloned into a pcDNA vector and expressed using the TnT Quick Coupled Transcription/Translation System (Promega). The absence of a specific band indicated that linc00261 is a transcript with no protein‐coding capacity. Luciferase in vitro translation served as positive control. (E) linc00261 was mainly located in the cytoplasm as shown by qRT‐PCR. (F) Prediction of linc00261 structure based on minimum free energy (MFE) and partition function. Color scale indicates the confidence for the prediction for each base with shades of red indicating strong confidence. (http://rna.tbi.univie.ac.at/). Figure S2 Expression of linc00261 was determined by northern blotting in 5 normal tissues and 5 paired GC tissues. [file JCMM-21-955-s001.doc]

**Materials and methods**

**Assessment of lncRNA protein-coding potential**

We determined whether this transcript has protein-coding potential using an in vitro translation assay and a combination of online softwares. For the in vitro translation assay, full-length linc00261 (amplified using primer linc00261 F2 and R2) was cloned into a pcDNA vector and expressed using the TnT Quick Coupled Transcription/Translation System (Promega). The absence of a specific band indicated that linc00261 is a transcript with no protein-coding capacity. Luciferase in vitro translation served as positive control. The online softwares include ORF Finder (http://www.ncbi.nlm.nih.gov/gorf/gorf.html), PhyloCSF (https://github.com/mlin/PhyloCSF/wiki) and Coding Potential Calculator (CPC; http://cpc.cbi.pku.edu.cn/).

**Subcellular fraction**

To determine the cellular localization of linc00261, cytosolic and nuclear fractions were isolated and collected with the Nuclear/cytoplasmic Isolation Kit (Biovision, USA) according to the manufacturer’s instructions. After that, total RNA were extracted from the collections of both cytoplasm and nucleus and cDNA was synthesized for the evaluation of linc00261. Briefly, we lysed SGC-7901 and AGS cells (provided by the Cell Bank of Chinese Academy of Science) with a buffer containing 10mM Tris-HCl (pH=7.4), 100mM NaCl, 2.5mM MgCl2, and 40mg/ml digitonin for 10min. The resulting lysates centrifuged with 2,060×g for 10min at 4°C. The supernatant was used for the cytosolic fraction. Subsequently, the pellets were washed and incubated with RIPA buffer at 4°C for 10 min. After centrifugation at 4°C for 10min at 2,060×g, the nuclear fraction was collected. RNAs extracted from each of the fractions were subjected to following RT-PCR analysis of the levels of nuclear control transcript (U6), cytoplasmic control transcript (GAPDH) and linc00261.

**Cell proliferation assays**

Cell proliferation assays were performed with Cell Counting Kit-8 (Dojindo, Japan) according to the manufacturer’s protocol. The transfected cells were seeded into 96-well plates at a density of 1×10^4^ cells/well. The absorbance in each well was evaluated by the 450 nm absorbance of reduced CCK-8 at the indicated time points. Three independent experiments were performed for each assay.

**Flow cytometric analysis**

For the cell cycle analysis, 1 × 10^6^ cells were seeded into each well of 6-well plates. Then cells were collected and fixed in chilled 70% ethanol at -20 °C for 1 h, followed by washing in phosphate-buffered saline (PBS) for three times. After fixation, cells were washed with cold PBS and stained with 0.5 ml of propidium iodide (PI) staining buffer, which contains 200 mg/mL RNase A, 50 μg/mL PI, at 37 °C for 30 min at 4℃ in the dark. The cell-cycle profiles were assayed at 488 nm on an EPICS 752 flow cytometer (Coulter, Hialeah, FL) equipped with MPLUS software (Phoenix 140 Flow Systems, San Diego, CA). Data were expressed as percentage distribution of cells in G0/G1, S and G2/M phases of the cell cycle.

For the apoptosis assay, cells were harvested and stained with Annexin V-fluorescein

isothiocyanate/ Propidium Iodide kit (KeyGen Biotech, China) according to the manufacturer’s instructions. The flow cytometry analysis was performed by FACSCalibur (BD Biosciences, USA). Results were the representative of three independent experiments with triplicate samples for each assay.

**Cell wound-healing and invasion Assay**

**For wound-healing assay, cells were seeded onto six-well dishes at 1×10^5^ cells per well. A single scratch wound was created using a p10 micropipette tip in to confluent cells. Cells were washed three times with PBS to remove cell debris, supplemented with assay medium, and monitored. Images were captured by phase-contrast microscopy at 0, 12 or 18 h after wounding.**

Cell invasion assay was carried out using 24-well inserts transwell chambers (8.0 mm, Corning, Corning, NY, USA) pre-coated with Matrigel. 5×10^4^ cells were suspended in 200 μL serum-free media and added into the upper chamber of an insert. The chambers were then incubated in cell culture medium with 10% FBS in the bottom chambers for 24 h before examination. The cells on the upper surface were scraped and washed away, whereas the cells on the lower surface were fixed with methanol and stained with 0.1% crystal violet. After that, crystal violet on the transwell membrane was dissolved in 33% acetic acid solution and measured the absorbance of

crystal violet (OD570 nm). Experiments were independently repeated in triplicate.

**SDS-PAGE and sensitive coomassie staining**

Protein samples (10 µL) after IP were run on 10% SDS-PAGE for 6 h at 4°C. The gel was disassembled and rinsed 3x 10 min. with deionized water. Proteins were fixed using 30 % ethanol, 2 % (w/v) phosphoric acid for 30 min. The gel was incubated in staining solution (0.02% Coomassie Brilliant Blue G250; 2% (w/v) phosphoric acid; 5% aluminiumsulfate; 10% ethanol) over night (or at least 3h). Then, the gel was destained with water and scanned for further analysis.

**Mass Spectrometry**

Differentially expressed protein bands were excised, washed with water, 40 mM ammonium bicarbonate / ethanol (1:1 v/v), reduced with 10 mM DTT and alkylated with 55 mM iodoacetamide. After alkylation, gel bands were repeatedly washed with 40 mM ammonium bicarbonate and ethanol, dehydrated with 100% acetonitrile and air-dried. Digestion with trypsin (Promega, Madison, USA; 10 ng/µl) was performed in 40 mM ammonium bicarbonate at 37°C overnight.

Tryptic peptides were analysed using a nanoAcquity UPLC system (Waters GmbH, Eschborn, Germany) coupled online to an LTQ Orbitrap XL mass spectrometer (Thermo Scientific, Bremen, Germany). Data were acquired by scan cycles of one FTMS scan with a resolution of 60000 at m/z 400 and a range from 300 to 2000 m/z in parallel with six MS/MS scans in the ion trap of the most abundant precursor ions. Instrument control, data acquisition and peak integration were performed using the Xcalibur software 2.1 (Thermo Scientific).

Using the MASCOT search engine (Matrix Science, London, UK; version 2.2.2), database searches were performed against the NCBI nr database (release 2012-08-11) with the human taxonomy. The significance threshold was *p*<0.01.

**RNA Pull-Down Assay**

Briefly, biotin-labeled RNAs were in vitro transcribed with the Biotin RNA Labeling Mix (Roche Diagnostics, Indianapolis, IN) and T7 RNA polymerase (Roche), treated with RNase-free DNase I (Roche), and purified with the RNeasy Mini Kit (Qiagen, Inc., Valencia, CA). One milligram of cell protein extract was then mixed with 50 pmol of biotinylated RNA biotin-labeled RNAs. Sixty microliters of washed streptavidin agarose beads (Invitrogen, Carlsbad, CA) were added to each binding reaction and further incubated at room temperature for 1 hour. Beads were washed briefly five times and boiled in sodium dodecyl sulfate buffer, and the retrieved protein was detected by the standard western blotting technique.

**RNA Immunoprecipitation (RIP)**

RIP experiments were performed using a Magna RIP RNA-Binding Protein Immunoprecipitation Kit (Millipore) according to the manufacturer’s instructions. The Slug antibodies were used for RIP (Abcam). The coprecipitated RNAs were detected by reverse transcription PCR and quantitative PCR. Total RNAs (input controls) and isotype controls were assayed simultaneously to demonstrate that the detected signals were the result of RNAs specifically binding to Slug (n=3 for each experiment).

**5**’**and 3’rapid amplification of cDNA ends (RACE) analysis**

We used the 5’- and 3’-RACE analyses to determine the transcriptional initiation and termination site of GCASPC using a SMARTer RACE cDNA Amplification Kit (Clontech, Palo Alto, CA, USA), according to the manufacturer’s instructions. Polymerase chain reaction (PCR) of the internal region was performed when starting points of 5’ and 3’ RACE had an unamplified gap. RACE PCR products were separated on a 1.5% agarose gel. Gel products were extracted with the Gel and PCR Clean-Up System (Promega, A9282), cloned into the pGEM-TVector Systems I (Progema, A3600) and sequenced bidirectionally using the M13 forward and reverse primers by Sanger sequencing at Invitrogen. At least five colonies were sequenced for everyRACE PCR product that was gel purified.

Supplemental Table 1. Correlation between linc00261 expression and clinicopathological characteristics of gastric cancer

| Clinical parameters | linc00261 expression | | χ*2 test* |
| --- | --- | --- | --- |
|  | Low no.cases | High no.cases | p-value |
| Age (years) |  |  | 0.544 |
| <50 | 26 | 10 |  |
| ≥50 | 29 | 15 |  |
|  |  |  |  |
| Gender |  |  | 0.755 |
| Male | 35 | 15 |  |
| Female | 20 | 10 |  |
|  |  |  |  |
| Size |  |  | 0.131 |
| ≥5 cm | 32 | 10 |  |
| <5 cm | 23 | 15 |  |
|  |  |  |  |
| Location |  |  | 0.249 |
| Distal | 27 | 10 |  |
| Middle | 21 | 8 |  |
| Proximal | 7 | 7 |  |
|  |  |  |  |
| Histologic differentiation |  |  | 0.211 |
| well/moderately | 19 | 11 |  |
| poorly/undifferentiated | 36 | 14 |  |
|  |  |  |  |
| Invasion depth |  |  | 0.006* |
| T1 | 10 | 7 |  |
| T2 | 16 | 15 |  |
| T3 | 18 | 3 |  |
| T4 | 11 | 0 |  |
|  |  |  |  |
| TNM stage |  |  |  |
| I | 11 | 15 | 0.003* |
| II | 13 | 5 |  |
| III | 23 | 5 |  |
| IV | 8 | 0 |  |
|  |  |  |  |
| Lymph node metastasis |  |  |  |
| Yes | 24 | 15 | 0.176 |
| No | 31 | 10 |  |
|  |  |  |  |
| Distant metastasis |  |  | 0.051 |
| Yes | 8 | 0 |  |
| No | 47 | 25 |  |

Differences among variable were assessed by chi-square test.*, the values had statistical significant differences.

Supplemental Table 2. Univariate and multivariate analyses of DFS in 80 GC patients by Cox regression analysis

| variable | DFS | | |
| --- | --- | --- | --- |
|  | Hazard ratio | CI (95%) | p value |
| Univariate analysis |  |  |  |
| Gender (male/Female) | 0.651 | 0.352-1.333 | 0.265 |
| Age (years,≤50/>50) | 0.685 | 0.312-1.360 | 0.254 |
| location (distal/middle+proximal) | 0.733 | 0.377-1.427 | 0.361 |
| Tumor size (cm,>5/≤5) | 1.118 | 0.576-2.172 | 0.742 |
| Histologic differentiation (well+moderately/poorly+undifferentiated) | 1.413 | 0.702-2.846 | 0.333 |
| Invasion depth (T3+T4/T1+T2) | 1.845 | 0.943-3.612 | 0.074 |
| lymph-node metastasis (Yes/No) | 3.247 | 1.475-7.143 | 0.003* |
| Distant metastasis (Yes/No) | 2.169 | 0.897-5.263 | 0.086 |
| TNM stage (III+IV/I+II) | 4.628 | 2.071-10.342 | <0.001* |
| linc00261 expression (low/high) | 2.242 | 1.101-4.976 | <0.001* |
|  |  |  |  |
| Multivariate analysis |  |  |  |
| TNM stage (III+IV/I+II) | 3.222 | 1.268–8.184 | 0.014* |
| lymph-node metastasis (Yes/No) | 1.754 | 0.719-4.292 | 0.517 |
| linc00261 expression (low/high) | 2.572 | 1.389-4.200 | 0.043* |

Supplemental Table 3. Linc00261 cDNA sequence.


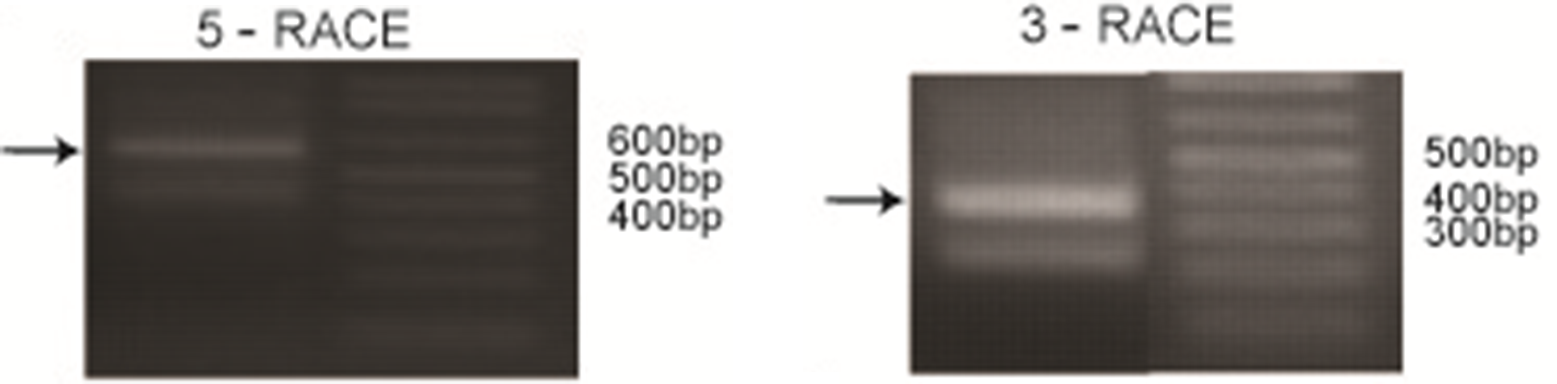


1 gctaaagtca acagtcgctt ggtttgagct caaataaatg cggggatgcc tcggctcgga

61 gcagtcggcc tcctccctcc gcgagctgga cgctccgcag cccgcccgcc agccggcccg

121 ccggccgccg caggaatccc tggataaaga ccagctcaac catcgctgag aaaacagacc

181 taggcttccc agggcggtta acccgccggc ctctgggcag agactacaaa acaaaataaa

241 acaacaacaa aaaactccca gtgtgtttcc tactcttctt tgtcttggag gaaagcaaag

301 ggagagaaat ggacttcacc agtggtcttt ggcttcatca attcacagga aatggcatca

361 agatggttca actaagacat gatcactaaa aacattataa taataccttt ttgaaaaact

421 cagtttctcc tgtttactaa atatttattt catcaacatg ggctgcgttc cactgtgtca

481 ggattctgca tgtgggtgga gcactgttcc agcctgagaa gatggttctg aggccactta

541 gcaagacatt ttccagcatg agcaggtttc tctgtggaaa tagtgacacc tgttctggtg

601 tgttgtcttt cctcagggaa cttaaggggt acaaagctcc tgaaaatgtt ctttatgctg

661 gttgaagctc ttatgtcgct gtactgattc cctacgatgc agatttgaat cacagagtaa

721 ttaaaatatg gatcaaataa ggctggggct caccaaggct gaaagctgta gccattcaag

781 gcatcatttc tgtcatgaaa atataggacc ttttcaaaac atgccttcag gaaggtgttc

841 tcttttcaaa caaaagtcta atgactgcat aactcttctt gaccacatct tacactttct

901 ctagacttgc ttatttacag ctactggaac aaaaagatga aatgaataaa gcacactgat

961 tgtcctaaga gtcttctgtg actaaaagca catgagcttc tccccaggct gagctttgac

1021 cctcccaagc atttccccaa aggccatgac actgcatcag attgctcctg gacactttcc

1081 tcagagtttt caacaaatat aaactgagga gtggagcaca gcaccagatt tgctaatcaa

1141 gaggcaatgg tcccagtcca aatgggtgct gtgtgtgaat acagagccca ggagaaggga

1201 aggaggtctg tgaacatcag gaattgcaaa atcttgctga accaatagac caacagccat

1261 taacatttgt caatgcaaag atcctaaaat atacaagtgt ggagttgtcc tgccctaaga

1321 gctgaagctg tgacaatcac gctgccatgg tagatattta agctcccctg aggttactga

1381 atctaacaat gaataaacaa tgaagagtag ctaatttttg gaaatctctt tctatgaccg

1441 gaaaatattc acaaactcag agtgattgtt tggggtgaaa gatggtccca tgccttgtgg

1501 gcctctcttt gctatctata caacgttgga aatgaacccc tcccttacag gaaagtgaac

1561 agcaaccaag gtgagcccac cctgggaggg cagagtgggt gtactttcct cgaagagccc

1621 tgaacacaac agggcacaca aacagccaca gcaccctcaa caatgcccca ccatctgccc

1681 accagggcac acagattcac gcagattcag tgacacattc caaaaaaagg gaattaagga

1741 ggacccatgg catggctaat ggggagagag ctttactact tttagcacca ttgctaatta

1801 ttggagcaaa gaaaggcata caaggaatgg aacccaacct gtttaaagta ataaatgtca

1861 accctaaagt gcatggaacc atctctcctt cccttttgag gactgtgtgc ttcacaagct

1921 gggcgtttgc tgcatggggt tttctaatgt aatgtgaatt ttcaatgtgc tttgtctctg

1981 tagagtggct ttaaaaagcc cttgaagtcc tcagctttaa atccatgcta ataggggaag

2041 tctttttttt ttttttttgg aggtgaggag ctttggttgg gagaaacatt gacccagact

2101 gcactctggg taaactaggg caaaagacct gatatcgtct gcagggtact tattaaccac

2161 tgttcatttg caggatcaat atttccagca aaggagtcca cactcaagat acactgctac

2221 gtctcacttt ttccttgaca caacaattta ttttataaat gcatctgctg gagaagaaaa

2281 ggcagtacca aggattgtgt cttttatctc caggatatgc aaagcgcatt tattttcagc

2341 ttttatctct ggaattgtat gttgatccca tacatataga ttgattttgt tcaggtagat

2401 aatgaaaagt caagtggaaa attacacaat ggcattcacg atcctagtct tatatcagta

2461 tataaattgg tttattattt cattctatta aagaggcttt tttttggtac caggaaaact

2521 tcaaaatgca ttgtgttgtc agcctcactg aattttcttt tggaggagga ggtagggagg

2581 ggaagccaaa gccccgattt ctcctgctcc ttttgctaat tgtattggct aaatgagctt

2641 tcacagaggg gggtcctggc tgctttttgc tctcccccag accacttgta ggttgcacac

2701 agggcttcta taactctttc tgagtcagga caaagatatc cgccgtgcac accctgtgac

2761 ataggtggat atttatttca tcccgggcaa ttctaaattt tgtcatgatg actgaaagag

2821 gcaatcccct cctgagcatt aagaggccag gggtgagagg gtggcagcat agaaaagcca

2881 ccacccagag acagcttgga gccatgatcc tggaggaaat gttattaata aacatctttt

2941 ggacatttgg tagcccgtgg agcttgctcc agcttaaagc aattaattca ggacactgac

3001 ataaactctc cagatctttg acagcttgga gccgaaataa aaatgctagt tctccgggga

3061 agagaagagg atggctagcc cttttctgga ctgtttgctt ataggcccag agagcaacct

3121 tccttgcaga atgaaatgca agggcacaaa caatgtacca gggagagaat agaagatggg

3181 aatgaacagt ttgaactatt tgctcagcag aagggcacca caatgctggg gtagtggttt

3241 tcccatgcgt ccactgtccc ttctttggga tcttttgttg tcccctgtct cctttggcca

3301 gataaaagca cagctgcagc attgcagata acaagtaggc cgtacttatc cccctaaatc

3361 tcggggtgcc tttactgtaa caagttatgc tcggctctct tttattttac aagaggatgg

3421 tgagggagag aatggaagaa cagagggggc ggacgtataa gacatttgac actgcctatg

3481 tctgatttct ctttctttct ttctttcttt ctttctttct ttctttcttt ctttcctttc

3541 tttctttctt gcaagctgtg atcggatgca atctttgtgg gacatttaaa tggaagggtt

3601 cattgatgtg tattgcttgc caagccaaaa tgttgccttt ggggaaaagg gagagaggtg

3661 ttcatggagt gcagggaaag gaggttttgg ggcagagatt ttgacttaaa ataaccagac

3721 ttcttctggc tgctgaaaag aggcaaaagt tttaaattgt caagtttaaa actatgttca

3781 gttatgattt gccacttctg aatattattt tggatttccc ctttcatgct tattttgttc

3841 agaatcctaa tgaatagagg ttgctggact cagggtaaaa gcaggatgaa ctggagatgg

3901 gacatacaag gtacttttgg aattgccata gattacacct ataatcagag taaatgtcat

3961 caacaaataa tcaaaatatt ttttacattt gctcttctaa aatcagagcc tattttaaat

4021 ataaaagaaa gtagatgtga taataatata aactacagtc acattaagtt gatattaaat

4081 tcaaaatcta acatagattt gcactgttgg gtgtgtgttc cgaatcagtg ggttttccca

4141 ctgatgttga tttcgggagc caggcttcaa tgtttaattc tattgtaatg tggttattta

4201 gcctgaatgg ttttataagg tggaaaggca aaaaatttaa ttccgaagaa aactagtgtt

4261 ttactatgac tgtggtaaac atttccaaag cccacctgtg ggaaatacaa agttttaatg

4321 ctgtgtgttt ttttgttttt gtattttgtc tcatcgacaa aactggcaga aaaaaacgct

4381 ttcgtatatt tttcctgctg ggtggtcaga aggaaaggcc gtgaagctaa aggtctccca

4441 ctgagacgct gttctgcaag gagccgacct cacgtgccgc cgccgccaga gaagagagca

4501 cctgttcatc tcggctcact gtgaggctga gctcagcgct ggcaggcgag gggccgcaag

4561 catcccccac agccaccgag agggcatccc tgcagggaaa tcatatccga catgcctgtg

4621 cccacagcag acttaagact gcctctaaaa tgtccatgaa gccattgtcc agtagagctg

4681 ttagttttaa caccagtgag tattaccttt ggttaaaagg atgcatgtgt ataggtgtat

4741 gtgtgtgcgt gtgtgtttgt gtttttggac ttgtgtggag aatgaagaaa gggttccatt

4801 taggcatttg caaatattcg atggcatcat gaaaagacaa aaaaatccta taaaatatat

4861 catattttgc tatgattttg tgtgtacatg taataaaatt attaagtata aaaaaaaaaa

4921 aaaa

Supplemental Table 4. Mass spectrometry analysis of the proteins pulled down by linc00261

| Hits | Description | protein score | protein mass (kDa) | PSMs | peptides | protein coverage % |
| --- | --- | --- | --- | --- | --- | --- |
| 1 | Heterogeneous nuclear ribonucleoprotein D0 | 8906.83 | 38.4 | 246 | 16 | 29.58 |
| 2 | snail family zinc finger 2 | 2304.56 | 34.5 | 85 | 12 | 34.02 |
| 3 | Heterogeneous nuclear ribonucleoprotein A/B | 1939.81 | 41.6 | 63 | 11 | 33.87 |
| 4 | Nucleolysin TIA-1 isoform p40 | 1680.23 | 38.9 | 52 | 7 | 21.08 |
| 5 | glycogen synthase kinase 3 beta | 1597.81 | 42.6 | 54 | 11 | 25.62 |
| 6 | DAZ-associated protein 1 | 1460.46 | 43.4 | 37 | 4 | 19.94 |
| 7 | Nucleolin | 1323.96 | 76.6 | 35 | 16 | 26.34 |
| 8 | Nuclease-sensitive element-binding protein 1 | 941.58 | 35.9 | 15 | 7 | 30.56 |
| 9 | RNA-binding motif protein, X chromosome | 723.96 | 42.3 | 14 | 5 | 13.55 |
| 10 | Y-box-binding protein 3 | 687.44 | 40.1 | 9 | 4 | 14.78 |


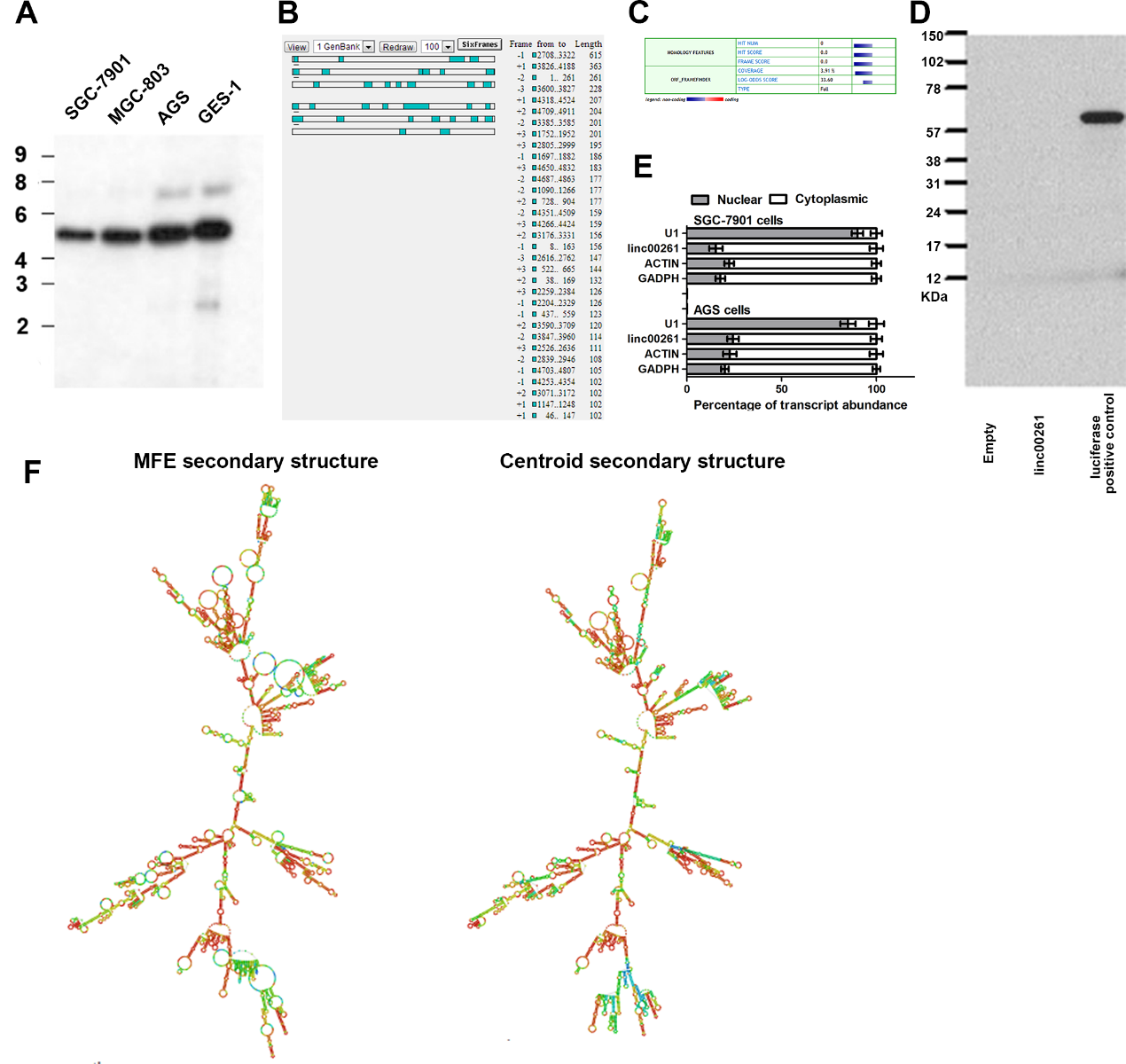


Supplemental Figure 1. (A) Northern blot analysis of linc00261 in GC cells. (B,C) linc00261 is a long non-coding RNA. Lack of open reading frame in the 4924-bp linc00261 sense sequence cloned from RACE, as verified by ORF Finder (B) and CPC (C). (D)In the translation assay, a 5598-bp genomic region containing linc00261 was cloned into a pcDNA vector and expressed using the TnT Quick Coupled Transcription/Translation System (Promega). The absence of a specific band indicated that linc00261 is a transcript with no protein-coding capacity. Luciferase in vitro translation served as positive control. (E) linc00261 was mainly located in the cytoplasm as shown by qRT-PCR. (F) Prediction of linc00261 structure based on minimum free energy (MFE) and partition function. Color scale indicates the confidence for the prediction for each base with shades of red indicating strong confidence. (<http://rna.tbi.univie.ac.at/>).


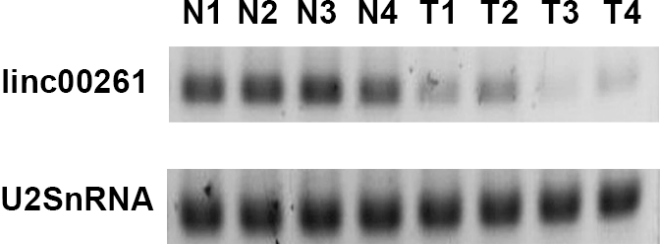


**Supplemental Figure 2.** Expression of linc00261 was determined by northern blotting in 5 normal tissues and 5 paired GC tissues.
